# Serverless Workflows for Containerised Applications in the Cloud Continuum

**DOI:** 10.1007/s10723-021-09570-2

**Published:** 2021-07-13

**Authors:** Sebastián Risco, Germán Moltó, Diana M. Naranjo, Ignacio Blanquer

**Affiliations:** Instituto de Instrumentación para Imagen Molecular (I3M), Centro mixto CSIC - Universitat Politècnica de València, Camino de Vera s/n, 46022 Valencia, España

**Keywords:** Cloud computing, Serverless computing, Workflow, Containers

## Abstract

This paper introduces an open-source platform to support serverless computing for scientific data-processing workflow-based applications across the Cloud continuum (i.e. simultaneously involving both on-premises and public Cloud platforms to process data captured at the edge). This is achieved via dynamic resource provisioning for FaaS platforms compatible with scale-to-zero approaches that minimise resource usage and cost for dynamic workloads with different elasticity requirements. The platform combines the usage of dynamically deployed auto-scaled Kubernetes clusters on on-premises Clouds and automated Cloud bursting into AWS Lambda to achieve higher levels of elasticity. A use case in public health for smart cities is used to assess the platform, in charge of detecting people not wearing face masks from captured videos. Faces are blurred for enhanced anonymity in the on-premises Cloud and detection via Deep Learning models is performed in AWS Lambda for this data-driven containerised workflow. The results indicate that hybrid workflows across the Cloud continuum can efficiently perform local data processing for enhanced regulations compliance and perform Cloud bursting for increased levels of elasticity.

## 1 Introduction

Cloud computing has become in the last decade the premier option for virtualised computing. It has increased hardware resource utilization and provided the ability to execute disparate computing workloads with complex requirements on shared computing infrastructures. Initial service delivery models, such as Infrastructure as a Service (IaaS), were exemplified by public Cloud services such as Amazon EC2 [4] and on-premises Cloud Management Platforms (CMPs) such as OpenStack [48]. These were later extended to accommodate additional models such as Platform as a Service (PaaS) and, more recently, Functions as a Service (FaaS). FaaS aims to rise the level of abstraction for application developers at the expense of relying on the infrastructure provider for automated elasticity, efficient virtual infrastructure provisioning and improved resource allocation.

Initial FaaS services, exemplified by public Cloud services such as AWS Lambda [6] and Azure Functions

S. Risco (✉) · G. Moltó · D. M. Naranjo · I. Blanquer
Instituto de Instrumentación para Imagen Molecular (I3M),
Centro mixto CSIC - Universitat Politècnica de València,
Camino de Vera s/n, 46022, Valencia, España
e-mail: serisgal@i3m.upv.es

G. Moltó
e-mail: gmolto@dsic.upv.es

D. M. Naranjo
e-mail: dnaranjo@i3m.upv.es

I. Blanquer
e-mail: iblanque@dsic.upv.es

[42], provide event-driven execution of functions coded in certain supported programming languages, offering automated resource allocation and ultra-elastic capabilities that superseded the ones found in traditional IaaS offerings. For example, a Lambda function can support up to 3000 concurrent executions which is two orders of magnitude beyond the default number of virtual machines that can be deployed in a newly created AWS account, which is 20 (and can of course be increased upon request). This could only be achieved by using lightweight virtualization technologies, as is the case of Firecracker [1] which allows deploying micro Virtual Machines (microVMs) in less than a second. In general, lightweight virtualization such as Linux containers (LXC) [39], introduced in 2008, also paved the way for this success. Indeed, the increased popularity gained by Linux software containers fostered the emergence of Docker [27] in 2013, which spawned an entire ecosystem of tools that boosted innovation and widespread adoption.

Both enterprise-based workloads and scientific computing benefited from this trend to provide encapsulated applications with all the dependencies to guarantee successful execution across a myriad of computing platforms. Docker images turned into a *de facto* approach for consistent multi-platform application delivery. The advent of containers, together with the sustained development of Container Orchestration Platforms (COPs) such as Kubernetes [15], paved the way for implementing the event-driven capabilities of FaaS under open-source platforms such as OpenFaaS [47], Knative [38] and Apache OpenWhisk [7]. These platforms mimic the functionality of public FaaS offerings for the execution of functions coded in certain languages within the premises of an organization. This computing paradigm for the Cloud, in which dynamic resource allocation is managed by the Cloud infrastructure provider, was coined as serverless computing [9].

However, the benefits of serverless computing cannot be restricted to just function-based computing, especially in the case of scientific computing [34] where complex software dependencies [36], resource-intensive requirements [49, 62] and, sometimes, the necessity of accelerated hardware is required [26], features that are not currently available in public Cloud serverless offerings. Currently, an AWS Lambda function cannot run beyond 15 minutes, use more than 10240 MBytes of RAM, use any accelerated computing device such as GPUs or use an ephemeral storage space greater than 512 MB, thus jeopardizing the adoption of these platforms by scientific computing.

Also, new challenges arise for scientific applications to harness the computing continuum, as indicated in the work by Beckman et al. [13], where they identify multiple infrastructures on which computing takes place such as interconnected sensors from IoT/Edge devices to computer clusters and Cloud infrastructures. Workflow-like applications may benefit from the orchestration of resources along the computing continuum. These applications may gather data at the edge, perform local processing to comply with privacy regulations on on-premises computing platforms and seamlessly profit from the elasticity of public Cloud infrastructures to reduce overall makespan.

Towards this vision, this paper introduces an architecture composed of open-source components that supports the execution of workflow-based data-processing applications packaged as Docker containers that can elastically provision resources from on-premises Clouds and perform automated bursting into a public Cloud using an event-driven serverless approach. The flexibility of this architecture provides a step forward in defining data-driven workflows that can execute along the Cloud continuum.

After the introduction, the reminder of this paper is structured as follows. First, Section 2 introduces the related work in the area of serverless scientific computing. Next, Section 3 describes the components of the designed platform and the Functions Definition Language created to support data-driven workflows along the Cloud continuum. Later, Section 4 describes a use case to assess the benefits of the developed platform that integrates Deep Learning models with serverless computing to produce cost-effective processing of videos from surveillance cameras to detect mask usage by the population. Finally, Section 5 summarizes the main achievements of the paper and points to future work.

## 2 Related Work

Several research groups understood from the early beginning that serverless computing could certainly benefit scientific computing. This is the case of the work by Jonas et al. [37] who introduced the PyWren tool to perform distributed computing using AWS Lambda, in order to support several programming models, building on the assumption that stateless functions can be a natural fit for data processing. Our earlier work in the area, MARLA (MapReduce on AWS Lambda)[1] by Giménez-Alventosa et al. [31] created a framework to execute Python-based MapReduce applications on AWS Lambda, thus producing a high-performant serverless open-source tool to execute High Throughput Computing (HTC) jobs without requiring any pre-provisioned computing infrastructure by the user. In this work we identified the unbalanced performance properties of serverless platforms such as AWS Lambda and produced a thorough research which identified performance variabilities in both network throughput and CPU performance for different invocations of the same Lambda function even with the same allocated resources. This sparked the need to create appropriate serverless load balancing strategies for HTC jobs that can minimise both execution time through proper dynamic load assignment thus resulting in reduced cost using the fine-grained billing models, as described in [32]. Moreover, the work by Fouladi et al. [30] also envisioned the mapping of thousands of parallel threads to multiple invocations of a Lambda function in order to achieve close to near-interactive completion times. They produced the *gg* software tool[2] which performs distributed compilation of large code bases, together with other use cases such as video encoding, offering an API with bindings for Python and C++.

The report by Sewak et al. [59] summarises the different applications of serverless computing along with the advantages and disadvantages of the main FaaS platforms in public Clouds, anticipating their growth and adoption in the near future, as well as indicating the need for new tools to harness the capabilities of these platforms and facilitating their adoption by developers. The applicability of serverless architectures to serve AI models has also been investigated in numerous studies. For example, the study carried out by Ishakian et al. [35] analyses the application of AWS Lambda to serve lightweight deep learning models, as the maximum available ephemeral storage in a function is 512 MB, concluding that such platforms can be suitable for workloads running on warm functions. However, their results show how cold starts can add significant overhead in latency times when compared to conventional services deployed on virtual machines. Aditionally, the papers conducted by Christidis et al. [23, 24] propose a set of optimisations for deploying machine learning workloads on serverless platforms. Some of these optimisations are in fact aligned with those implemented in our work, such as minimizing container images or loading them on the ephemeral storage of functions in order to overcome the maximum size of the deployment package. These studies further conclude that it is worth adapting such applications to serverless platforms in view of the potential savings and robust elasticity, and point to the growing need to support specialised AI-accelerated hardware on such platforms.

Indeed, supporting serverless computing for scientific computing requires solving specific challenges that lie ahead the development of our early prototype. To begin with the first challenge, a problem that remains unsolved is chaining function composition to produce serverless workflows that can fully exploit resources from on-premises to public Clouds including computing at the edge for local data preprocessing. The serverless trilemma by Baldini et al. [10] identified that engineering function composition for a serverless application is possible but function composition must obey a substitution principle with respect to synchronous invocation and invocations should not be double-billed, what poses additional constraints to enact serverless workflows with respect to traditional workflow systems.

An early work by Malawski [40] explored the idea of serverless workflows for processing background tasks of Web applications and how to rethink serverless architectures for executing scientific workflows, introducing a prototype based on Google Cloud Functions coupled with the Hyperflow workflow engine. In this line, the work by Skluzacek et al. introduced Xtract, a service to process large collections of scientific files to extract metadata from various file types. They used funcX [20] to develop the prototype, a federated FaaS system to enable function execution across heterogeneous distributed resources. These functions are snippets of Python code and the system relies on Globus transfer to perform data staging. The authors found that it can be difficult to modify

[1]MARLA - https://github.com/grycap/marla

[2]gg - https://github.com/StanfordSNR/gg applications for stateful execution, since the state is not easily shared among functions. Thus, poorly designed solutions may lead to significant communication overhead.

Despite the large number of open-source FaaS frameworks, few research has been dedicated to serverless workflows, specially to those that are inherently data-driven because they require processing data across multiple stages of the workflow. For example, Faas-flow [58] provides function composition for the OpenFaaS framework by creating chains of functions that can be executed both synchronously and asynchronously with support for parallel execution with branching, even upon certain conditions. Other workflow engines that run on top of Kubernetes may be used to provide some support for serverless workflows. This is the case of Argo Workflows [8], an open-source container-native workflow engine for orchestrating parallel jobs on Kubernetes which models multi-step workflows as sequences of tasks via DAGs (Directed Acyclic Graphs) and which provides support for event-driven workflow automation.

In fact, serverless workflows is an active research area where several contributions are being proposed. For example, the work by Ristov et al. [57] introduces a language to describe function choreographies to connect serverless functions. Indeed, the Serverless Workflow Specification (SWS) [25] was recently approved as a Cloud native Sandbox level project to define declarative workflow models that orchestrate event-driven serverless applications. We expect that this specification will bring benefits in the area of consistency, providing a common way of describing serverless workflows, portability and accessibility, to provide interoperability among serverless workflow runtimes. However, for the time being, this specification allows to compose a workflow from pre-existing serverless functions and, therefore, does not involve function provisioning. Techniques such as *Dynamic parallelism* supported by AWS Step Functions are beneficial for the orchestration of microservices-based applications. Nevertheless, this technique is mainly employed for control-driven workflows, where the connections between the activities or tasks in a workflow represent a transfer of control from the proceedings task (or tasks) to the one (or ones) that follow [60]. However, the focus of our work is on data-driven workflows where a task input depends on the output data generated by the previous task.

Concerning the support to the computing continuum, several authors have previously explored this topic. For example, the work by Balouek-Thomert et al. [11] presents a vision to enable such a computing continuum and they set the focus on enabling edge-to-cloud integration to support data-driven workflows. They focus on stream-oriented workflows to filter data near the sources but they do not use a serverless approach and no open-source implementation is provided. The work by Baresi et al. [12] introduces the A3-E unified model for the Mobile-Edge-Cloud continuum which exploits the FaaS model to bring computation to the continuum. It uses Apache OpenWhisk to support the implementation together with AWS Lambda. However, no support for workflows is introduced.

It is precisely at the verge of this state-of-the-art that lies this contribution, producing an open-source platform that provides serverless scientific computing along the Cloud continuum, including both on-premises and public Clouds, and that supports data-driven workflow enactment in serverless platforms and multi-Cloud hybrid deployments of infrastructures. To best of the author's knowledge, this is the first platform that supports event-driven serverless scientific computing simultaneously harnessing resources from multiple Clouds (exemplified in our case via OpenStack and AWS Lambda). The platform, together with the definition of the use case described in this paper has been released as open-source, publicly available in GitHub, for the sake of reproducibility.

## 3 Components to Support Serverless Workflows along the Cloud Continuum

This section identifies the main components employed to support hybrid serverless workflows that can span across on-premises Clouds and public Cloud platforms to process data that may be captured at the edge. First, the SCAR[3] [50] software for serverless scientific computing in public Clouds is described. Later, the open-source OSCAR[4] [53] framework to support serverless computing for data-processing applications in on-premises Clouds is covered. Finally, this section introduces the Functions Definition Language

[3]SCAR - https://github.com/grycap/scar

[4]OSCAR - https://github.com/grycap/oscar

(FDL) created to define the functions together with its relationship with data-driven serverless computing workflows.

The main contribution of this paper lies in the development of a new version of the OSCAR framework to match the same computing model provided by SCAR. This allowed the integration of both components to support the same computing model across both on-premises and public FaaS platforms for data-processing applications. Another key contribution is the development of a novel FDL to define data-driven serverless workflows that can execute along the Cloud continuum, in order to support the definition of use cases that require processing at different levels of this continuum. Notice that, by building on existing open-source software that is being used in production we aim to foster long-term sustainability of the developed architecture.

### 3.1 SCAR: Serverless Scientific Computing in Public Clouds

SCAR is an open-source framework that supports a High Throughput Computing model [52] to create embarrassingly parallel event-driven file-processing serverless applications on public FaaS platforms, currently supporting AWS Lambda. The applications can be packaged as Docker images that can be optionally stored in Docker Hub [28] (alternative means include Amazon S3). This allows to execute complex scientific applications in AWS Lambda, thus being able to spawn up to 3000 parallel invocations (depending on the region used). There are strict computing requirements per invocation in AWS Lambda, which are currently 10240 MB of RAM, 512 MB of ephemeral storage that is potentially shared across invocations and 15 minutes of execution time. Therefore, this typically requires using container minimization strategies in order to fit the Docker container within AWS Lambda's runtime environment, such as those available in tools like *minicon* [33], which analyses an application execution to obtain a filesystem that exclusively contains the dependencies detected.

For those applications that do not fit within AWS Lambda's computing requirements, SCAR provides a seamless integration with AWS Batch [5] an elastic-cluster as a service offering by AWS which dynamically deploys a cluster in charge of executing jobs packaged as a Docker images and which can grow and shrink depending on the number of jobs queued up at the Local Resource Management System (LRMS). This integration allows to delegate into AWS Batch functions invocations that require longer execution times, larger amount of memory or even GPU resources for accelerated execution, as described in the work by Risco et al. [56].

However, there are applications that can benefit from the event-driven behaviour of serverless platforms but that require strict privacy requirements and, therefore, cannot be run in a public Cloud provider. Also, there are organizations that are already operating an on-premises Cloud managed by a Cloud Management Platform such as OpenStack [48] and, therefore, do not want to spend additional economic cost from provisioning resources from a public Cloud. The OSCAR platform described in the following section was developed to support these scenarios.

### 3.2 OSCAR: Open-Source Serverless Computing for Data-Processing Applications

OSCAR is an open-source platform to support the Functions as a Service computing model for compute-intensive applications. OSCAR can be automatically deployed on multi-Clouds in order to create highly-parallel event-driven file-processing serverless applications that execute on customized runtime environments provided by Docker containers than run on an elastic Kubernetes cluster that grows and shrinks depending on the usage of resources.

The automated deployment of an OSCAR cluster on multi-Clouds is achieved using:

- IM (Infrastructure Manager) [16], a TOSCA-compliant [46] Infrastructure as Code (IaC) tool to deploy complex customized virtualised infrastructures on the major on-premises and public Infrastructure as a Service providers.
- CLUES (CLUster Elasticity System) [3], an elasticity manager that allows virtual clusters to grow and shrink in terms of the number of nodes. It has plugins for popular systems such as Kubernetes, Apache Mesos, SLURM, etc.
- EC3 (Elastic Cloud Computing Cluster) [17], which combines the two developments above to deploy automated self-scaling clusters on multi-Clouds.

An OSCAR cluster features the integration of the following components:

- Kubernetes [15], a container orchestration platform, thus managing containerised applications across multiple hosts. It provides basic mechanisms for deployment, maintenance, and scaling of applications.
- OpenFaaS [47], an open-source FaaS framework to execute short-lived functions on top of a container orchestration platform.
- MinIO [43], an open-source object storage system with Amazon S3's API compatibility.
- OSCAR, the component in charge of creating a function together with the required resources to support event-driven batch-based GPU-aware executions on top of the Kubernetes cluster for serverless scientific computing.

The creation of an OSCAR function allows users to upload files to the object storage system which triggers the execution of the function to perform the data-processing, with automated elasticity if it is required, and the output data is stored in any of the object storage systems supported. This is the case of Onedata [29] a global data management system that provides access to distributed storage resources for data-intensive scientific computations. This is used to support EGI DataHub, a federated data storage layer auspiced by EGI (European Grid Infrastructure), the largest federated Cloud in Europe. OSCAR is compatible with the EGI DataHub. Other object storage systems, such as Amazon S3, can be employed to store the output data, thus allowing to trigger the AWS Lambda functions.

Figure 1 describes the internal architecture of an OSCAR cluster. The bottom part depicts a horizontally elastic Kubernetes cluster that is deployed via EC3 from pre-defined TOSCA templates that are employed by the Infrastructure Manager (IM) to provision and configure the front-end node of the cluster. This node is configured with the required Kubernetes services, the CLUES elasticity manager, and a private instance of the IM server deployed in the aforementioned front-end node. This way, the clusters become autonomous in deciding whether to scale out (provision additional nodes from the underlying Cloud) or to scale in, depending on the number of pods that are pending to be executed.

The upper part of the figure shows the main components of the cluster together with a typical workflow. For this contribution, OSCAR was completely redesigned in order to support the computing model offered by SCAR. To this aim, OSCAR exposes a secure REST API that receives requests to create functions. It is responsible for creating the corresponding input and output buckets in MinIO, depending on the function configuration, and configure the event notifications in order to trigger the function execution upon a file upload to the input bucket. OpenFaaS is employed in order to perform synchronous executions of function invocations, typically short-lived, which is the most common use case of serverless computing. However, in order to support resource-intensive event-driven scientific computing, asynchronous executions are required. To this aim, OSCAR creates a Kubernetes job for each asynchronous invocation that are delegated into the Kubernetes workload scheduler for efficient execution. These jobs are wrapped with the FaaS supervisor,[5] an Input/Output data manager especially created for multi-cloud settings, which allows to gather data from input data storages and upload output data into the corresponding data storages. The supported data storages are depicted in the right part of the picture.

Security has been addressed using best practices depending on the infrastructure being employed. For example, Lambda functions use pre-defined IAM (Identity and Access Management) Roles[6] that follow the Principle of Least Privilege (PoLP) so that they can only access the resources required, such as an Amazon S3 bucket to store the generated output data. The deployment via EC3 of the Kubernetes cluster dynamically generates a token for the user to connect to the OSCAR web-based user interface and tokens to access the Kubernetes dashboard and MinIO browser, in case the user wants to directly access them. Dynamic generation of secrets prevents from reusing passwords that would cause severe security implications such as unauthorized access breach that could be exploited for nefarious purposes. Moreover, the OSCAR API requires basic auth and is exposed through a Kubernetes ingress that supports SSL. Finally, Onedata leases tokens, which can be revoked at any time, in order to provide access to the space.

[5]FaaS Supervisor - https://github.com/grycap/faas-supervisor

[6]IAM Roles: https://docs.aws.amazon.com/IAM/latest/UserGuide/id_roles.html

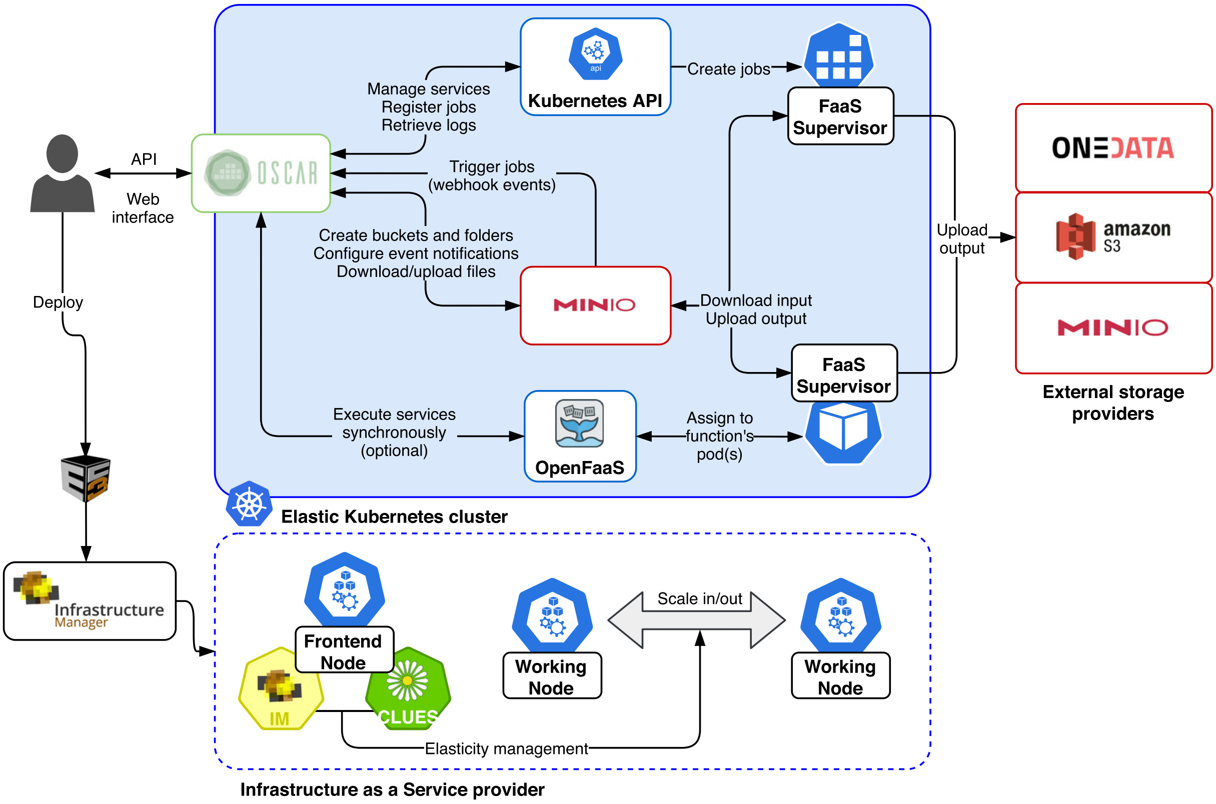

**Fig. 1** Architecture of the OSCAR platform and interactions among their services

### 3.3 Functions Definition Language for Data-Driven Serverless Workflows

To support the deployment of data-driven workflows of serverless functions that require complex data-processing, we opted for defining a YAML-based Functions Definition Language (FDL) that specifies the requirements for each function and how they are linked. Notice that, unlike the Serverless Workflow Specification (SWS) which provides a workflow definition out of existing FaaS functions, our language focuses on the definition of the functions to be dynamically created across the hybrid Cloud. Therefore, our proposal could be coupled at a later stage with the SWS language to provide a portable, interoperable description of workflows out of the dynamically created functions from our platform.

Two top-level resources are defined in a FDL (see a sample document in Fig. 2, used to support the case study described in Section 4):

- Functions, which are created in a Cloud provider and they are assigned a name, a certain amount of computing resources together with a shell-script that will be executed, as part of the function invocation, inside a container created out of a specific Docker image that may available in Docker Hub. The function will be triggered whenever a file is uploaded to a specific folder within a storage provider and the shell-script will be in charge to perform the data processing on the file.
- Storage Providers, which become sources of events for input data processing and store the output data results from a function invocation. By using as output from a function the input storage provider from another function, a precedence relationship is established among them and a data-driven link is created.

Notice that our platform focuses on data-processing applications and, therefore, each function is linked

```
---
functions:
  aws:
  - lambda:
      name: scar-mask-detector
      memory: 1024
      init_script: mask-detector.sh
      container:
        image: grycap/mask-detector-yolo:mini
      input:
      - storage_provider: s3
        path: scar-mask-detector/intermediate
      output:
      - storage_provider: s3
        path: scar-mask-detector/result
  oscar:
  - my_oscar:
      name: oscar-anon-and-split
      memory: 2Gi
      cpu: '1.0'
      image: grycap/blurry-faces
      script: blurry-faces.sh
      input:
      - storage_provider: minio
        path: oscar-anon-and-split/input
      output:
      - storage_provider: s3.my_s3
        path: scar-mask-detector/intermediate
storage_providers:
  s3:
    my_s3:
      access_key: xxxxxx
      secret_key: xxxxxx
      region: us-east-1
```

**Fig. 2** Functions Definition Language file to deploy the workflow used in the following section to an input storage provider (so that the function is invoked upon a file upload) and also to one or more output storage providers (where the output file results of the processing will be stored). The choice of Docker allows to have complex execution environments that may be required by scientific applications, which typically rely on multiple libraries, require specific OS distributions, etc. This way we can have a consistent execution environment whether using a public Cloud or an on-premises one. Note that SCAR's ability to handle Docker images as runtime environments within both AWS Lambda and AWS Batch also supports the decision to use Docker images in this environment.

The choice of shell-scripts instead of providing bindings for specific programming languages responds to the goal of supporting scientific applications, which are typically legacy applications that are unfeasible to be adapted to other programming languages other than those initially used to code them. Also, this allows to execute *any* application that supports the command-line, thus broadening the scope of applications that can be supported, instead of forcing developers to adapt their legacy applications to a certain programming language.

The integration of SCAR and OSCAR tools, together with the adaptation of the FaaS Supervisor, has been carried out in order to support these new FDL files. For this purpose, as previously indicated, OSCAR has been redesigned to support the function definition model through its REST API, along with other improvements such as a refreshed web interface and the ability to retrieve execution logs for enhanced visibility. The SCAR tool has also been improved to allow communication with OSCAR endpoints, as well as a parser update to handle the new functions definition files. Finally, the FaaS Supervisor required minor changes to support the loading of storage providers' credentials from the new files. In this way, as shown in Fig. 3, SCAR has become a tool capable of orchestrating resources to support file-processing serverless computing along the Cloud continuum. Hence, SCAR manages the creation of the required resources both in AWS and in the OSCAR cluster together with the corresponding output folders in the Onedata space. The composition of the different steps of the workflow is achieved by using the output bucket of one function as the input of the subsequent one. Therefore, to start the execution of the workflow, users only have to upload a file to the input bucket of the first function.

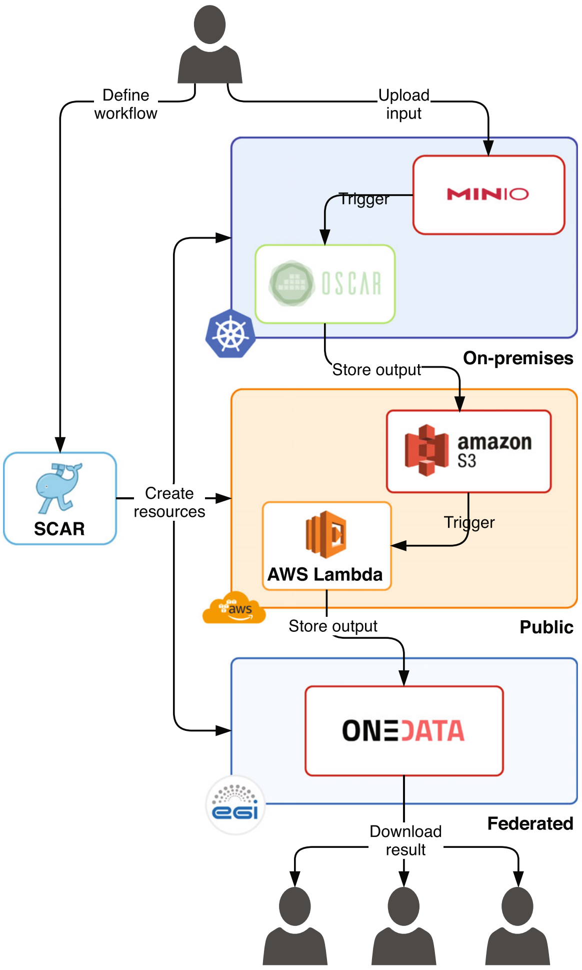

**Fig. 3** Simplified diagram of a hybrid serverless workflow that involves public, on-premises and federated cloud resources

Note that infrastructure management is being left out from the FDL, in line with the serverless approach of delegating in the Cloud provider for this task. In our case, it is at deployment time of the OSCAR cluster when the user/administrator indicates the maximum number of nodes of the cluster, together with their computing requirements. This way, the user can focus on the definition of the application workflow and let the cluster auto-scale within the on-premises Cloud. In the case of functions created with SCAR, the concurrency limits can be specified by the user at creation time.

In order to demonstrate the benefits of the designed platform, the following section introduces a use case related to public health in smart cities.

## 4 Use Case: Mask Wearing Detection via Anonymised Deep Learning Video Processing

Data analysis in the context of smart cities is an active area of research and the role of data processing (Big Data) to extract knowledge from dense networks of sensors across a city is summarised in the review work by Nuaimi et al. [2], in the work of Camero et al. [18] and in the work by Chen et al. [22]. As an example, the work by Bello et al. [14] highlighted the importance of sound as a source of information about urban life, focusing on monitoring noise pollution and audio surveillance. Indeed, the work by Spadini et al. [61] focused on ambient sound processing across smart cities in order to detect abnormal events such as gunshots, sirens, etc.

Using a similar approach, we focus in this study on smart camera networks [55], which are distributed systems that perform computer vision tasks using multiple cameras. These have implications in activities such as surveillance with cameras that capture the natural movement of individuals and vehicles in everyday environments, as indicated in the work by Chen et al. [21].

This use case targets an scenario of video surveillance in which it is required to provide increased monitoring capabilities for the authorities to take better public health decisions. With the COVID-19 global pandemic that affected the entire world starting late 2019, many national authorities have regulated the mandatory use of face masks in order to minimize the spread of the virus across the population. To this aim, this use case introduces a workflow entirely based on open-source components that allows to determine the people that are not wearing a mask out of sampled images from video recordings that could be obtained from a network of cameras distributed throughout a city. This may allow public health authorities to better devote resources to minimize this trend in the specific areas being monitored.

However, according to the NIST Guide to Protecting the Confidentiality of Personally Identifiable Information (PII) [41], a person's face is considered a PII because it can unequivocally identify a human being. Therefore, in order to protect the privacy of the individuals, a pre-processing stage is performed in order to blur the faces before applying a deep learning model to perform the face mask recognition. This is why a hybrid serverless workflow is required so that processing is performed along the Cloud continuum, where data is captured at the edge (camera devices), pre-processing is carried out in an on-premises Cloud for regulatory compliance purposes and, finally, processing and storing of final results is carried out in a public Cloud using a serverless platform for increased elasticity and long-term persistence.

The steps of the workflow can be shown in Fig. 4. A set of cameras from a smart camera network periodically take short videos that are automatically uploaded to an on-premises Cloud with a MinIO installation. Each uploaded video triggers an event that starts the "Anon and split" function within the OSCAR cluster in order to extract a frame every 5 seconds of video and perform the initial anonymisation phase on the extracted images to blur the faces using the Blurry-Faces tool [44]. This phase takes an average of 65 seconds to chunk and anonymise 1 minute of video at a resolution of 1920x1080. Therefore, considering the computational requirements and the need to comply with the local regulations related to the use of PII, this phase can be performed in the on-premises Cloud.

The resulting anonymised images are then uploaded to an Amazon S3 bucket in order to start the inference process in the public Cloud. Each uploaded image triggers the "Mask detector" Lambda function responsible for using the *face-mask-detector* [54] Deep Learning model in order to compute the percentage of people in the picture that are not wearing a face mask. The output images are made available in another Amazon S3 bucket to guarantee long-term data persistence and for the responsible stakeholder

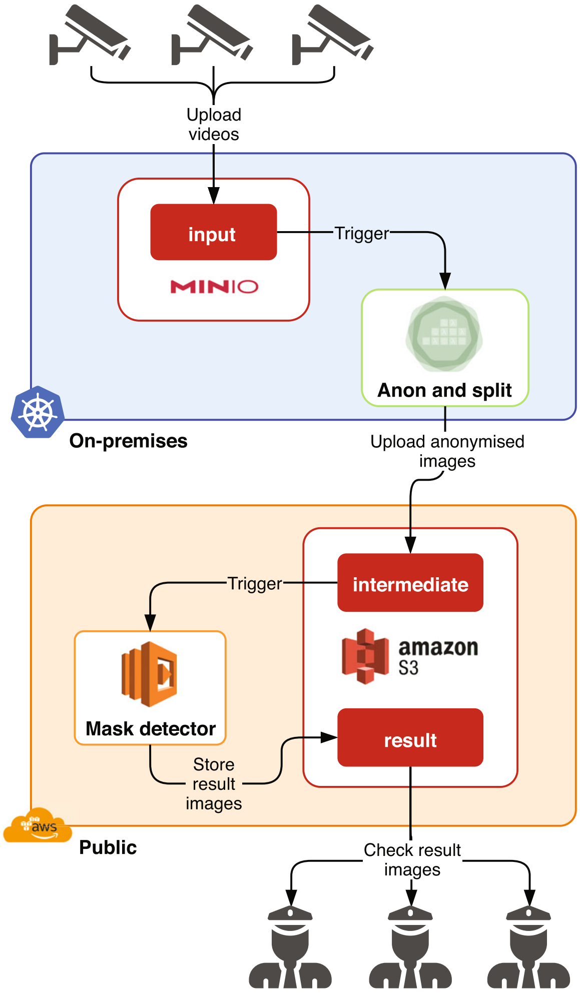

**Fig. 4** Workflow for the defined use case involving face mask detection on anonymised images on a hybrid Cloud to take actions upon the results obtained. We rely on Amazon S3 instead of EGI DataHub to store the output data for the sake of easier reproducibility. A sample image result of the processed workflow is shown in Fig. 5.

Notice that this technology can be applied by the local authorities to perform a quantitative and systematic evaluation of the fulfillment of the regulations to determine if further enforcement is required. The ability to safely outsource the embarrassingly parallel part of the workflow to a public Cloud supports the scalability of the designed approach.

The following subsections describe the approach employed to create the workflow, together with optimization techniques applied for increased cost-effectiveness.

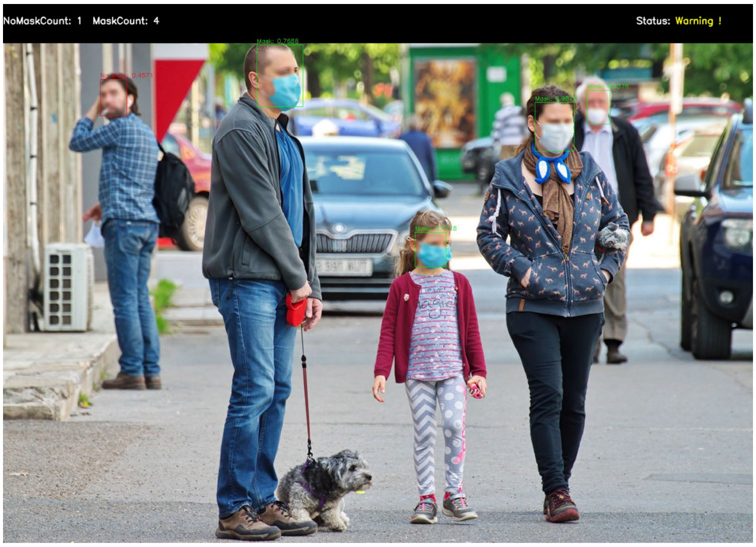

**Fig. 5** Result image that differentiates people not wearing face masks

### 4.1 Optimal Resource Allocation for the Lambda Function

The allocation of computing resources for an AWS Lambda function is linearly dependent on the amount of allocated RAM. Hence, increased amount of RAM may reduce the execution time but, at the same time, increases the cost, which is billed in milliseconds of execution time. Therefore, in order to choose the optimal amount of memory to achieve cost-efficient executions in a timely manner, we relied on AWS Lambda Power Tuning [19], an open-source tool to optimize Lambda functions for cost/performance using a data-driven approach. The tool performs the execution of the function with different memory allocations in order to compute both the execution time and the total cost.

Figure 6 shows the output results obtained by the aforementioned tool showing average values ($N$ = 5 repetitions) for the execution cost and the execution time for the mask detector function running with different RAM values starting at 256 MB, the least amount of RAM required to execute the deep learning model, until 4096 MB. Larger memory amounts have been omitted since the execution time remained at similar values, as can be seen in the line between 2048 MB and 4096 MB, while the cost kept increasing. Note that the first invocation for each memory amount is performed when the container image is not available in the AWS Lambda environment, which results in an increased execution time due to the cold start, while in the remaining ones (i.e. when the Docker image is already available for the function invocations) no variation is appreciated. The function was created using SCAR in the *us-east-1* region and outside of a VPC (Virtual Private Cloud), which provides faster execution times.

The figure shows that the best cost is obtained with the least amount of memory, at the expense of achieving the worse (maximum) execution time. Notice that the execution time reduces almost linearly when using up to 512 MB of RAM. From this point on, increasing the RAM, which proportionally affects the CPU

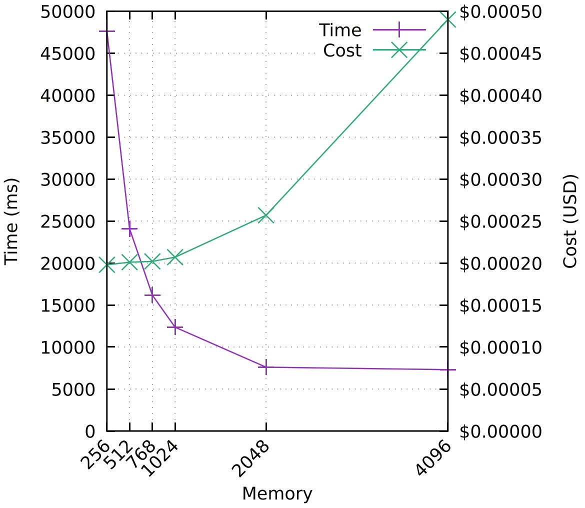

**Fig. 6** Time and cost analysis for the mask detector function running on AWS Lambda allocation, provides moderate improvements in the execution time with an increased cost, which starts to grow considerably from 1024 MB upwards. Finally, allocating 4096 MB provides a marginal improvement with respect to 2048 MB at the expense of a substantial cost increment. For this particular application, the optimal amount of memory lies between 768 and 1024 MB of RAM, depending on the budget restrictions of the user.

Several key results are obtained from this analysis. On the one hand, allocating additional memory to a Lambda function tends to reduce the execution time, but this is not always the case (compare the execution time with 2048 and 4096 MB of RAM in the figure above). On the other hand, the execution time is billed in milliseconds and, therefore, optimization is a mandatory strategy when creating a Lambda function. Marginal optimizations on a Lambda function that are executed a large amount of times end up in producing significant cost savings.

### 4.2 Case Study Design

Two scenarios were designed in order to prove the benefits of the designed platform. In the first one, the whole workflow is executed on the on-premises platform. In the second one, the proposed hybrid workflow has been used, which involves resources both from the on-premises Cloud and the public Cloud. This allows to better assess the benefits of adopting a hybrid approach along the Cloud continuum.

The on-premises platform employed to conduct the experiment consists of an elastic Kubernetes cluster deployed with the EC3 tool, since the use of an Infrastructure as Code (IAC) [45] approach allows to guarantee deterministic provisioning of customized virtual infrastructures. The cluster is composed of a front-end node and a maximum of 5 working nodes, each one with 4 vCPUs and 8 GB of memory. The underlying infrastructure is a physical cluster supported by OpenStack which includes 14 Intel Skylake Gold 6130 processors, with 14 cores each, 5.25 TB of RAM and 2 x 10GbE ports and 1 Infiniband port in each node.

For the “Anon and split” function, which always runs on the OSCAR platform, 1 vCPU and 2 GB of memory were set. Note that although the working node instances have 4 vCPU, the internal components of Kubernetes require a small amount of resources (0.2 vCPU and 250 MB approximately), which makes the 4 entire vCPUs unavailable for processing. Therefore, the Kubernetes scheduler can only assign for execution three function jobs simultaneously on the same working node. Moreover, the “Mask detector” function was executed on the public or on-premises Cloud depending on the scenario. Considering the results of the study carried out in the section 4.1, 1024 MB of RAM were chosen for this function in both scenarios, setting 1 vCPU when running in the on-premises OSCAR cluster.

The SCAR client was used to perform the workflow deployment, depicted in Fig. 4 across the hybrid infrastructure using the FDL file shown in Fig. 2. This file shows the definition of two functions, one in AWS Lambda and one in the OSCAR cluster, together with their computing requirements. It also indicates the Docker image from which a container will be created to execute the script that will process the file that triggers the execution of the function. Decoupling the infrastructure provisioning with the workflow deployment allows to reuse the underlying provisioned infrastructure to support multiple hybrid workflows from different users, thus supporting a multi-tenant approach.

To perform the different tests, a sample video with a resolution of 1920x1080 pixels and a duration of 186 seconds was used. After the “Anon and split” phase this resulted in 37 images to be processed by the “Mask detector” function. The different experiments conducted on each scenario together with the results obtained are presented below.

### 4.3 Results and Discussion

This section analyses the results obtained from the execution of the different scenarios previously defined.

#### *4.3.1 Video Processing Analysis*

In order to prove the effectiveness of hybrid serverless workflows for processing data produced at the edge, the times obtained after 5 workflow runs to process a single video were measured. As mentioned above, the first function is responsible for extracting frames from the input video every 5 seconds and then applying an anonymisation strategy based on distorting the faces. This “Anon and split” function will always run on the on-premises platform (OSCAR), which will ideally be deployed on private Cloud infrastructures that comply with established data protection regulations, or even on intermediate devices located near the edge (fog computing). Afterwards, the "Mask detector" function will be triggered by each image resulting from the previous function, hence it will be possible to evaluate its performance when processing several images in parallel on both scenarios.

Figure 7 shows the times obtained for each function after processing the sample video 5 times in the two defined scenarios. The first function required an average time of 206.4 seconds on the first scenario and 227.4 seconds on the second one. This increment is caused by the uploading of the images to the input bucket of the second function, which in the first case was located in the same cluster (due to the use of MinIO), while in the hybrid workflow is on Amazon S3, so the files must be uploaded via the Internet.

In the second function, a significant improvement can be seen due to the massive parallelism supported by AWS Lambda, which allows all the images to be processed in parallel. The average time obtained in the first scenario was 111.4 seconds, since the maximum level of parallelism was 3 processing jobs within a single node. Notice that to process a single video, the CLUES elasticity manager of OSCAR does not have enough time to scale out the cluster by deploying additional working nodes. In AWS Lambda, however, the processing of all the images completed in an average of 19.6 seconds, which supposes an improvement of

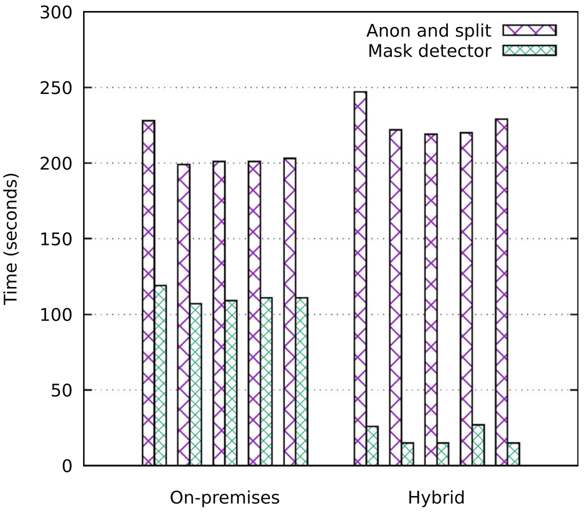

**Fig. 7** Execution times of the workflow functions in the two defined scenarios

82.4%. As you may observe, the processing time of the 27 images in AWS Lambda is longer than that of a single image, i.e. not all are strictly performed in parallel. This is due to the fact that the time has been measured from the moment the first image starts to be processed until the result of the last image is saved. Delays in uploading files to S3 from the first function directly affect the total image processing time, which causes that not all the images are uploaded simultaneously. Therefore, good network connectivity between the two Clouds will considerably increase the performance of the second function.

Moreover, as observed in the figure, the first execution of each function has longer times than the rest. This can be explained by the fact that these values are measured when the function is triggered for the first time on the platform. On the one hand, in the OSCAR Kubernetes cluster, the first time a function is executed on a working node, the container image must be downloaded from Docker Hub, which generates a delay of up to 13% in the first function and 10% in the second one. Notice that the image used in the first function is considerably larger (1.51 GB) than that used in the second function (219 MB), as it has been reduced by *minicon* to fit on the constrained environment of AWS Lambda. On the other hand, the "Mask detector" function in the second scenario is performed on AWS Lambda and, as described in [51], if the function is "cold", the time taken to download the container image must be added to the time taken to start up the corresponding execution environment, increasing the gap from the average value.

Also, the execution cost of the "Mask detector" function in the second scenario has been analysed. As discussed in the Section 4.1, the optimal performance point for the function in AWS Lambda was approximately 1024 MB of RAM. The average billed time after a cold start of the function is 12908 ms, which translates into an image processing cost of $0.00021556. On the other hand, the subsequent executions, having the image of the container available, will be much faster, obtaining an average billed time of just 2123 ms and a cost of $0.00003545. Summarizing, the processing cost of the 37 images generated by the video can range from $0.00131165, when the function is "warm", to $0.00797572 in the worst case, if all the executions were triggered exactly at the same time and none of them had the container image in the file system.

The total times measured when performing a complete workflow execution are shown in Fig. 8. As mentioned above, the maximum values in each scenario, displayed in the box chart as outliers, match the first invocations of each function when the container images are not present in the working node and, in the case of AWS Lambda, the function is cold. Despite the fact that these situations considerably increase the time needed to complete the workflow, they can easily be avoided by downloading the images when each node in the cluster is started. For AWS Lambda several techniques exist to keep the functions "warm", such as invoking them periodically or activating the *Provisioned Concurrency* feature, at the expense of increasing the cost of the application.

In an edge computing scenario, on-premises clusters used by the sensors (the surveillance cameras in the experiment) will be of reduced scale so they cannot scale up to the sizes of AWS Lambda. Moreover, automatic scaling up the cluster through CLUES would require booting up resources and for this specific case will imply a prohibitive overhead. Although the figures of the experiment with a larger-scale on-premise cloud would have been more competitive with respect to the AWS Lambda, they would not be comparable in a large-scale production-level scenario.

### *4.3.2 Parallel Video Processing Analysis*

In order to test the scalability of the designed system, we executed the workflow under the two scenarios with several number of videos to be processed. The videos were uploaded simultaneously to the input bucket thus simulating the data capturing process from several cameras at the edge. Figure 9 shows the results obtained. Notice that the benefits of performing a hybrid approach appear since the beginning, as identified in the previous section, but the margin of improvement increases as the workload increases. Indeed, the impressive elasticity capabilities of AWS Lambda, that may perform up to 3000 parallel invocations greatly surpasses the bottlenecks that are typically found in on-premises Clouds where the parallel execution slots are limited to those available in the provisioned infrastructure.

At a more detailed level, the workflow execution for the processing of a single video on the first scenario has only been executed on one node. Despite the fact that CLUES triggers the scale-out order to the 4 remaining working nodes shortly after the creation of the 37 Kubernetes jobs for treating the images, all the jobs ended up on the active WN before the new ones completed their start up and configuration process. This means that resources are wasted when the load is low, as the platform is able to scale, but not in time. Thus, after a period of idle time the new nodes are shut down again. As can be seen, when the on-premises platform receives more load (starting from 5 videos), the processing time is reduced in both scenarios as a result of the availability of more resources for parallel job processing.

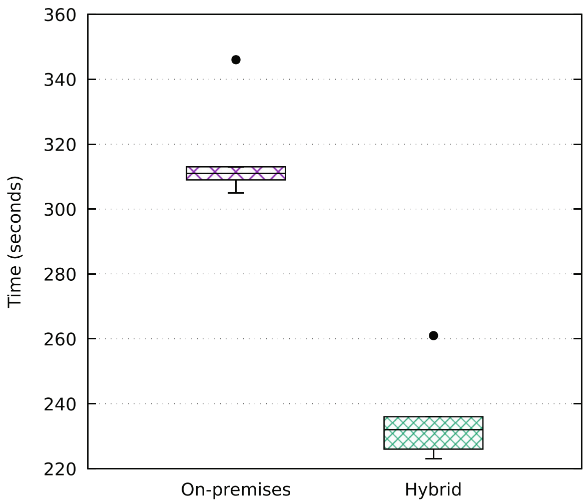

**Fig. 8** Total execution times of the workflow in the two defined scenarios

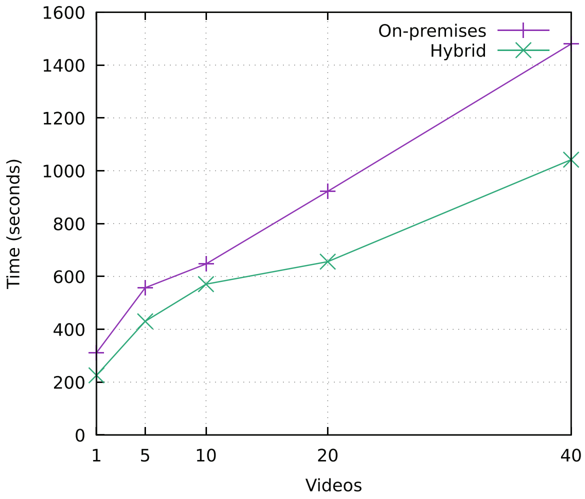

**Fig. 9** Measured times after processing different amounts of videos in parallel

It is important to point out that the gap between the two lines in Fig. 9 can be closed by increasing the number of parallel execution slots and reducing the time until they are initially available for execution. The former is bounded by the underlying physical hardware available, which is fixed, and the allocation of resources to the deployed Virtual Machines, i.e., the instance types, which can be configured at deployment time. The latter depends on the elasticity rules employed in the cluster. In our case, the CLUES elasticity manager governing the rules to scale out (add additional nodes) and scale in (terminate the free nodes) was configured to only start the front-end of the cluster and a working node in charge of performing the job executions. This is a conservative strategy that aims to minimize energy consumption in an on-premises Cloud and only reactively provision additional resources whenever they are needed. Since CLUES rules can be configured, the user may prefer to have a pre-provisioned fleet of VMs that are immediately available upon moderate changes in the workload to be processed.

The use of an open-source stack that can be fully configured by the user in order to seamlessly perform both infrastructure provision along the Cloud continuum and data-driven workflow enactment using a serverless approach is an important step forward in widespreading the adoption of this techniques for scientific computing. Traditional serverless use cases focused on unpredictable bursts of short-lived requests, as is the case of web applications. However, we have demonstrated that compute-intensive, workflow-based applications can also benefit from the event-driven capabilities and automated resource management provided by serverless computing.

## 5 Conclusions

This paper has introduced an open-source platform that supports the definition of event-driven file-processing workflows that can execute across the Cloud computing continuum that features underlying elasticity in the provisioning of resources. The ability to Cloud burst into a public Cloud using a serverless approach introduces an unprecedented level of elasticity when compared to traditional approaches based exclusively on Virtual Machines.

The seamless integration between SCAR, which supports the execution of containers within AWS Lambda to bring serverless for scientific computing, and OSCAR, which provides the FaaS computing model for file-processing applications on Kubernetes clusters, has allowed to create hybrid data processing workflows across the Cloud continuum. These workflows can orchestrate automated provisioning of resources both in the on-premises Cloud, through elastic Kubernetes clusters and in the public Cloud, through the use of serverless services such as AWS Lambda.

A use case based on smart camera networks with applications in smart cities for video surveillance has been envisaged and assessed, in order to efficiently determine the usage of face masks across the population out of processed videos using Artificial Intelligence models. The use case has been made publicly available in GitHub[7] in order to guarantee its reproducibility. The experiments show that it is affordable and efficient to deviate a computing intensive part of the processing to AWS Lambda, rather than processing it on limited-scale, on-premises clusters, even if those clusters would have better network connectivity. This fact is more evident as the scale factor increases

Future works include dynamic resource orchestration across the Cloud-to-Things continuum, where the workflow can anticipate the expected incoming workload in order to further adapt the resources. This would minimize the amount of time invested in provisioning additional nodes within the on-premises Cloud and the cold-start incurred by the Lambda functions once they have scaled-to-zero. We also plan to adapt OSCAR to minimalistic Kubernetes distribution to move part of the event-driven functionality of OSCAR closer to the edge, by enabling it to run on IoT devices, allowing the composition of workflows that begin the processing on the data gathering device itself.

**Acknowledgements** The authors would like to thank the European Union for the project "Artificial Intelligence in Secure PRIvacy-preserving computing coNTinuum" (AI-SPRINT), with code 101016577, funded under the H2020 Framework Programme and also the regional government of the Comunitat Valenciana (Spain) for the project IDIFEDER/2018/032 (High-Performance Algorithms for the Modeling, Simulation and early Detection of diseases in Personalized Medicine),

[7] Mask detector workflow - https://github.com/grycap/scar/tree/master/examples/mask-detector-workflow co-funded by the European Union ERDF funds (European Regional Development Fund) of the Comunitat Valenciana 2014–2020.

**Funding** Open Access funding provided thanks to the CRUE-CSIC agreement with Springer Nature.

**Open Access** This article is licensed under a Creative Commons Attribution 4.0 International License, which permits use, sharing, adaptation, distribution and reproduction in any medium or format, as long as you give appropriate credit to the original author(s) and the source, provide a link to the Creative Commons licence, and indicate if changes were made. The images or other third party material in this article are included in the article's Creative Commons licence, unless indicated otherwise in a credit line to the material. If material is not included in the article's Creative Commons licence and your intended use is not permitted by statutory regulation or exceeds the permitted use, you will need to obtain permission directly from the copyright holder. To view a copy of this licence, visit http://creativecommons.org/licenses/by/4.0/.
